# Pan-cancer analysis of the TRAF family genes and their correlation with prognosis, TME, immune and drug sensitivity

**DOI:** 10.1186/s40001-024-01875-8

**Published:** 2024-06-02

**Authors:** Bin Yao, Weikang Hu, Yu Chen, Jing Li, Kuirong Jiang, Jin Dou

**Affiliations:** 1 Changshu NO.2 People’s Hospital, Changshu, China; 2 Pancreas Center, The First Affiliated Hospital of Nanjing Medical University, Nanjing, China; 3 Huai’an Hospital Affiliated to Yangzhou University, Huai’an, China; 4 The Affiliated Huai’an Hospital of Xuzhou Medical University, Huai’an, China; 5 Medical College, Yangzhou University, Yangzhou, China

**Keywords:** Tumor necrosis factor receptor-associated factors (TRAFs), Pan-cancer analysis, Tumor microenvironment (TME), Immune, Cancer prognosis, Drug sensitivity

## Abstract

**Background:**

Tumor necrosis factor receptor-associated factors family genes play a pivotal role in tumorigenesis and metastasis, functioning as adapters or E3 ubiquitin ligases across various signaling pathways. To date, limited research has explored the association between tumor necrosis factor receptor-associated factors family genes and the clinicopathological characteristics of tumors, immunity, and the tumor microenvironment (TME). This comprehensive study investigates the relationship between tumor necrosis factor receptor-associated factors family and prognosis, TME, immune response, and drug sensitivity in a pan-cancer context.

**Methods:**

Utilizing current public databases, this study examines the expression levels and prognostic significance of tumor necrosis factor receptor-associated factors family genes in a pan-cancer context through bioinformatic analysis. In addition, it investigates the correlation between tumor necrosis factor receptor-associated factors expression and various factors, including the TME, immune subtypes, stemness scores, and drug sensitivity in pan-cancer.

**Results:**

Elevated expression levels of tumor necrosis factor receptor-associated factor 2, 3, 4, and 7 were observed across various cancer types. Patients exhibiting high expression of these genes generally faced a worse prognosis. Furthermore, a significant correlation was noted between the expression of tumor necrosis factor receptor-associated factors family genes and multiple dimensions of the TME, immune subtypes, and drug sensitivity.

**Supplementary Information:**

The online version contains supplementary material available at 10.1186/s40001-024-01875-8.

## Introduction

Tumor necrosis factor receptor-associated factors (TRAFs) comprise a group of cytoplasmic adaptor proteins that play diverse roles in mammalian physiological processes [1]. To date, this family includes six classical members (TRAF1–6) and one atypical member (TRAF7) [2, 3]. Notably, the classical members are characterized by a conserved amino acid sequence known as the TRAF domain, located at the C-terminal end. This domain is crucial for their interaction with various receptors and signaling proteins, integral to the function of TRAF proteins [4]. Except for TRAF1, all members of the TRAF family exhibit E3 ubiquitin ligase activity, attributed to a homogeneous RING finger domain at the N-terminal, essential for this activity [5, 6]. Consequently, TRAFs serve dual functions as both adaptors and E3 ubiquitin ligases. They interact with a broad spectrum of receptors, including TNF receptors (TNFRs), Toll-like receptors (TLRs), transforming growth factor-β (TGF-β) receptors, and receptors for interleukins (IL-1, IL-2) and interferons (IFN). These interactions are crucial in regulating cellular processes such as proliferation, differentiation, survival, apoptosis, and immune responses [7–11]. Numerous signal transduction pathways are implicated in tumor pathogenesis. For instance, elevated expression of TRAF1 is linked to the advancement of B lymphocyte malignancies, such as chronic lymphocytic leukemia (CLL), non-Hodgkin lymphoma (NHL), and Burkitt’s lymphoma. This progression is primarily due to TRAF1's interaction with TNFRs like CD30 and the Epstein–Barr virus (EBV) protein LMP1, which leads to the activation of the NF-κB signaling pathway [12–15].

In light of the absence of comprehensive research on the association between TRAF family genes and cancer, this review delves into the clinical characteristics and prognostic significance of TRAF family genes (TRAF1–7) in pan-cancer samples, utilizing data from The Cancer Genome Atlas (TCGA). In addition, the study synthesizes insights into the relationship between TRAF expression and the TME, immune subtypes, and drug sensitivity in cancer patients.

## Materials and methods

### Differential expression of TRAF family genes in human pan-cancer tissues

To identify variations in the expression of TRAF family genes across different cancers, we utilized RNA sequencing data (FPKM format) along with clinical characteristics, survival information, immune subtypes, and stemness scores (based on DNA methylation and RNA) for 33 cancer types. This data was sourced from TCGA database and accessed via the University of California Santa Cruz (UCSC) Xena data set platform (http://xena.ucsc.edu/) [16]. Comprehensive details of the 33 cancer types, encompassing abbreviations, full names, the quantity of cancerous and normal samples, and other relevant data, are presented in the Supplementary table (Table S1). We employed Perl software for data organization and to extract expression levels of TRAF family genes. The Wilcoxon test method was applied to assess the variations in expression between cancerous and adjacent normal tissues across different cancer types [17]. Significance levels are denoted as “*”, “**”, and “***”, corresponding to *P* values of < 0.05, < 0.01, and < 0.001, respectively. Furthermore, we employed the 'ggpubr' R package within R software for boxplot generation, 'pheatmap' for heatmap creation, and 'corrplot' for analyzing correlations within the TRAF family. Notably, data sets with fewer than five normal samples were excluded from the final analysis to minimize their impact on the results.

### Survival analysis based on TRAF family gene expression in human cancer

Survival information for each case was extracted from the TCGA database to explore the relationship between TRAF gene expression and clinical outcomes. Survival rates were calculated using the Kaplan–Meier method, and differences were assessed with the log-rank test, considering *P* values < 0.05 as statistically significant. The median expression level of each TRAF family gene served as the threshold for categorizing cancer cases into high-risk and low-risk groups. To illustrate survival probabilities, we utilized the “survminer” and “survival” packages in R to generate survival curves based on these risk categories. Furthermore, Cox proportional hazards analysis was conducted to elucidate the relationship between TRAF family gene expression and cancer prognosis. Subsequently, forest plots summarizing these associations were created using the 'survival' and 'forestplot' packages in R.

Moreover, the link between TRAF family gene expression and overall cancer survival was corroborated using the Kaplan–Meier plotter (https://kmplot.com/analysis/) and PrognoScan (http://dna00.bio.kyutech.ac.jp/PrognoScan/index.html) online databases. These platforms were employed to further assess the association with clinical outcomes, including overall survival (OS), disease-specific survival (DSS), disease-free survival (DFS), and recurrence-free survival (RFS).

### Analysis of TRAF family genes expression correlation with immune subtypes, TME, and stemness score in pan-cancer tissues

For this analysis, we sourced data on immune subtypes, stromal and immune cell scores, and stemness scores from the UCSC database. These data sets were then processed using the “estimate” and “limma” packages in R to determine the correlation between TRAF family genes expression and these critical cancer-related parameters. Correlation analyses were performed to assess the relationship between TRAF family gene expression and both RNA stemness score (RNAss) and DNA stemness score (DNAss), using Spearman's method with the “cor.test” function and the “limma” package in R. In addition, the interaction of TRAF family gene expression with the TME and stemness scores in selected cancers was explored using the “reshape2”, “ggpubr”, “ggplot2”, and “limma” packages in R. The processed drug sensitivity data were sourced from the CellMiner database.

(https://discover.nci.nih.gov/cellminer/). Subsequent data analyses and visualization of results were conducted using the “imput”, “limma”, and “ggplot2” packages in R.

### RT–qPCR analysis

For RNA extraction, TRIzol Reagent (Life Technologies, Carlsbad, CA, USA) was employed to isolate total RNA from 40 paired pancreatic adenocarcinoma (PAAD) and adjacent non-tumorous tissues, following the manufacturer's protocol. Subsequent spectrophotometric quantification was performed, and 1 μg of the total RNA was utilized in a 20 μl reaction for reverse transcription (RT) using the iScript cDNA Synthesis Kit (Bio-Rad, Hercules, CA, USA), as the manufacturer's instructions. Human 18S rRNA expression served as a normalization control for the initial mRNA concentration in the tissues. The expression levels of the target genes were quantified using the 2-ΔCT method. Primer sequences used in this study are detailed in Supplementary table S5.

### Functional experiments

#### Clone formation assay

Cells were seeded in six-well plates at a density of 800 cells per well and incubated in complete medium for a period of 2 weeks. Following incubation, the cells were treated with 0.1% crystal violet solution (Beyotime, China) for 30 min, followed by a wash with phosphate-buffered saline (PBS). Colonies with a diameter exceeding 1 mm were subsequently counted.

#### Wound healing assay

Cells were seeded in 6-well plates at a density of 8 × 105 cells per well. When density reached approximately 100%, a straight scratch was made using a 200-µl pipette tip. The loose cells were removed by washing with PBS, and phase images were taken by inversion fluorescence microscopy. ImageJ software was used to measure the relative wound areas.

#### Transwell assay

Approximately 4 × 104 MiaPaca-2 and PANC-1 cells were uniformly seeded into the upper layer of each Transwell membrane, and culture medium (750 μl) containing 10% foetal bovine serum was used as a chemoattractant to induce cell migration to the other side. After incubating at 37 °C under an atmosphere with 5% CO2 for 24 h, the cells above the membrane were gently wiped off using cotton-tipped swabs, while the cells that passed through the membrane were stained with 0.1% crystal violet for 30 min to assess cell migration. Finally, representative images from five random views were obtained under a microscope. Matrigel (BD Bioscience Pharmingen) was spread on the upper layer to assess cell invasion according to the manufacturer’s protocol, and the remaining procedure following the steps described above.

### Statistical analysis

For comparing two groups, Student's *t* test was employed, while one-way ANOVA or the Kruskal–Wallis test was utilized for analyses involving more than three groups. Survival outcomes were assessed using log-rank tests, and the results were visualized through Kaplan–Meier survival curves. The Spearman correlation analysis was applied to investigate the associations of TRAF family gene expression with the TME, stemness scores, immune subtypes, and drug sensitivity. The Cox proportional hazards model was used to identify independent prognostic factors. *P* < 0.05 was deemed indicative of statistical significance.

## Results

### Expression and correlation of TRAF family genes

**Fig. 1:**
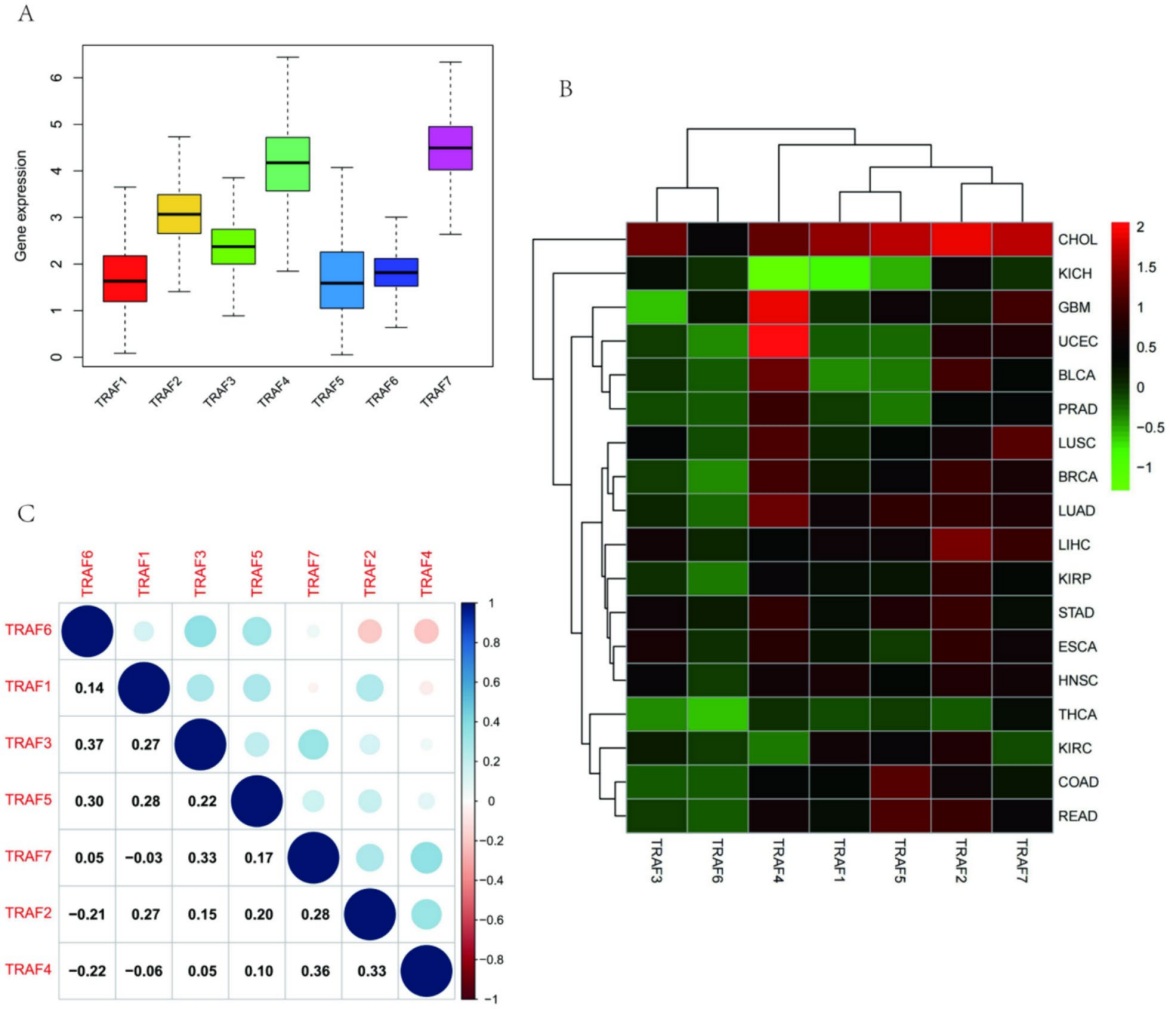
Expression and correlation of TRAF family genes across various cancers. **A** Depicts the expression levels of TRAF family genes in different tumor types. **B** Showcases the specific expression levels of TRAF family genes in each cancer type, with red indicating overexpression and green indicating underexpression. **C** Correlations among TRAF family genes, where blue signifies positive correlation and red signifies negative correlation. The size of each circle is proportional to the absolute value of the correlation coefficient

The analysis of 33 cancer types revealed that TRAF2, TRAF3, TRAF4, and TRAF7 are predominantly overexpressed, whereas TRAF1, TRAF5, and TRAF6 exhibit lower expression levels (Fig. 1A). Detailed examination indicated that the highest expressions of TRAF1, TRAF2, TRAF3, TRAF5, and TRAF7 were in Cholangiocarcinoma (CHOL), while TRAF4 was most expressed in Uterine Corpus Endometrial Carcinoma (UCEC) and TRAF6 in Glioblastoma multiforme (GBM) (Fig. 1B). Furthermore, the study investigated the interrelations within the TRAF gene family. A significant positive correlation was observed between TRAF3 and TRAF6 (Correlation coefficient = 0.37, Fig. 1C), whereas TRAF4 showed a notable negative correlation with TRAF6 (Correlation coefficient = − 0.22, Fig. 1C).

**Fig. 2:**
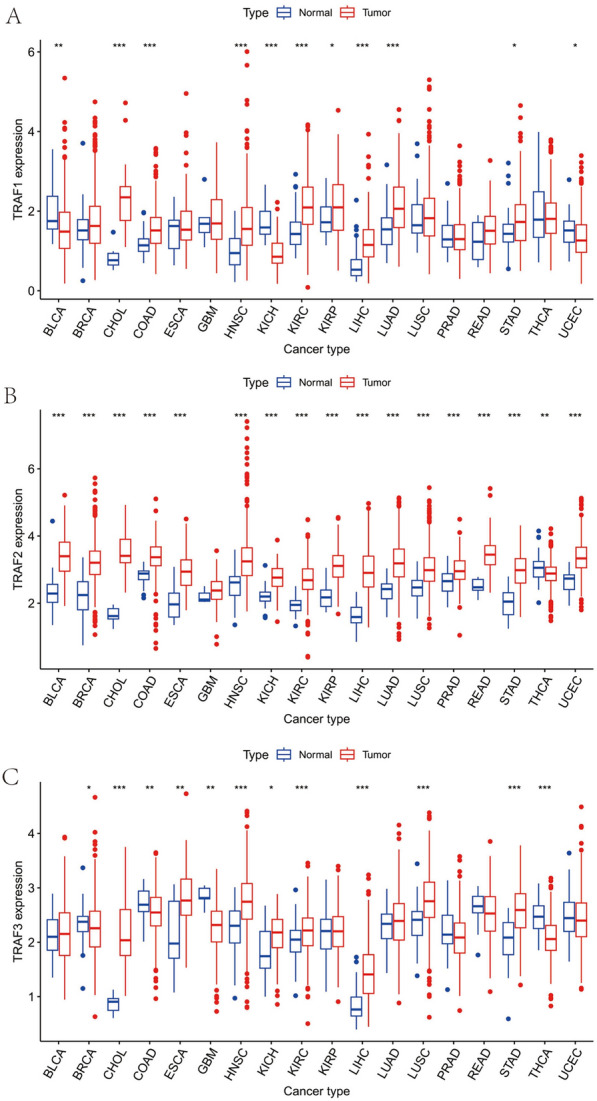
Differential expression of TRAF family genes in pan-carcinoma and para-carcinoma. **A** TRAF1. **B** TRAF2. **C** TRAF3. Red represents tumour and blue indicates normal tissue. (*P < 0.05, **P < 0.01 and ***P < 0.001)

We sourced TRAF family gene expression RNA sequencing data (TCGA data) from the UCSC database, specifically examining TRAF1 expression across various cancer types (Table 1). Our analysis revealed elevated TRAF1 expression in several cancers, notably CHOL, colon adenocarcinoma (COAD), head and neck squamous cell carcinoma (HNSC), kidney renal clear cell carcinoma (KIRC), kidney renal papillary cell carcinoma (KIRP), liver hepatocellular carcinoma (LIHC), lung adenocarcinoma (LUAD), and stomach adenocarcinoma (STAD). Conversely, reduced TRAF1 expression was observed in Bladder urothelial carcinoma (BLCA), Kidney chromophobe (KICH), and Uterine corpus Endometrial carcinoma (UCEC) (Fig. 2A). Our investigation further identified that TRAF2 demonstrated increased expression in a variety of cancer types, including BLCA, breast invasive carcinoma (BRCA), CHOL, COAD, esophageal carcinoma (ESCA), HNSC, KICH, KIRC, KIRP, LIHC, LUAD, LUSC, prostate adenocarcinoma (PRAD), rectum adenocarcinoma (READ), STAD, and UCEC. However, a diminished expression of TRAF2 was observed in thyroid carcinoma (THCA) (Fig. 2B). In addition, our data revealed that TRAF3 was more prominently expressed in cancers, such as CHOL, ESCA, HNSC, KICH, KIRC, LIHC, LUSC and STAD. In contrast, lower levels of TRAF3 expression were noted in BRCA, COAD, GBM, and THCA (Fig. 2C). Elevated expression of TRAF4 was observed in a range of cancer types, including BLCA, BRCA, CHOL, COAD, ESCA, GBM, HNSC, KIRP, LIHC, LUAD, LUSC, PRAD, READ, STAD, and UCEC. In contrast, lower expression levels of TRAF4 were noted in KICH and KIRC (Supplementary Fig. 1A). Furthermore, TRAF5 showed increased expression in BRCA, CHOL, COAD, GBM, HNSC, KIRC, LIHC, LUAD, LUSC, READ, and STAD, while its expression was reduced in BLCA, KICH, PRAD, and UCEC (Supplementary Fig. 1B). In addition, TRAF6 exhibited higher expression in CHOL and STAD, but lower expression in BLCA, BRCA, COAD, KIRP, LUAD, LUSC, PRAD, THCA, and UCEC (Supplementary Fig. 1C). Finally, TRAF7 was more highly expressed in BLCA, BRCA, CHOL, COAD, ESC), GBM, HNSC, KIRP, LIHC, LUAD, LUSC, PRAD, READ, STAD, THCA, and UCEC, with a reduced expression in KIRC (Supplementary Fig. 1D).

**Table 1:** Differential expression of TRAF family genes in different cancer types

Gene	Expression	Cancer types	Survival rate	Cancer types
TRAF1	High	CHOL, COAD, HNSC, KIRC, KIRP, LIHC, LUAS, STAD,	Increased	HNSC, MESO, PAAD, SKCM
	Low	BLCA,KICH, UCEC	Decreased	COAD, KIRC, LGG, THYM
TRAF2	High	BLCA, BRCA, CHOL, COAD, ESCA, HNSC, KICH, KIRC, KIRP, LIHC, LUAD, LUSC, PRAD, READ, STAD, UCEC	Increased	STAD
	Low	THCA	Decreased	ACC, COAD, LGG, LIHC, MESO
TRAF3	High	CHOL, ESCA, HNSC, KICH, KIRC, LIHC, LUSC, STAD	Increased	LGG, PAAD,
	Low	BRCA, COAD, GBM, THCA	Decreased	ACC, LIHC, THYM, UVM
TRAF4	High	BLCA, BRCA, CHOL, COAD, ESCA, GBM, HNSC, KIRP, LIHC, LUAD, LUSC, PRAD, READ,STAD, UCEC	Increased	BLCA
	Low	KICH, KIRC	Decreased	ACC, HNSC, KIRC, LIHC, SARC
TRAF5	High	BRCA, CHOL, COAD, GBM, HNSC, KIRC, LIHC, LUAD, LUSC, READ, STAD	Increased	BLCA, UCEC
	Low	BLCA, KICH, PRAD, UCEC	Decreased	ACC, KIRC, KIRP, LGG, LIHC
TRAF6	High	CHOL, STAD	Increased	KIRC, READ
	Low	BLCA, BRCA, COAD, KIRP, LUAD, LUSC, PRAD, THCA, UCEC	Decreased	
TRAF7	High	BLCA, BRCA, CHOL, COAD, ESCA, GBM, HNSC, KIRP, LIHC, LUAD, LUSC, PRAD, READ, STSAD, THCA, UCEC	Increased	
	Low	KIRC	Decreased	KIRC, LGG, LUSC, OV, PAAD, SKCM

### Prognostic value of TRAF family genes in pan-cancer

**Fig. 3:**
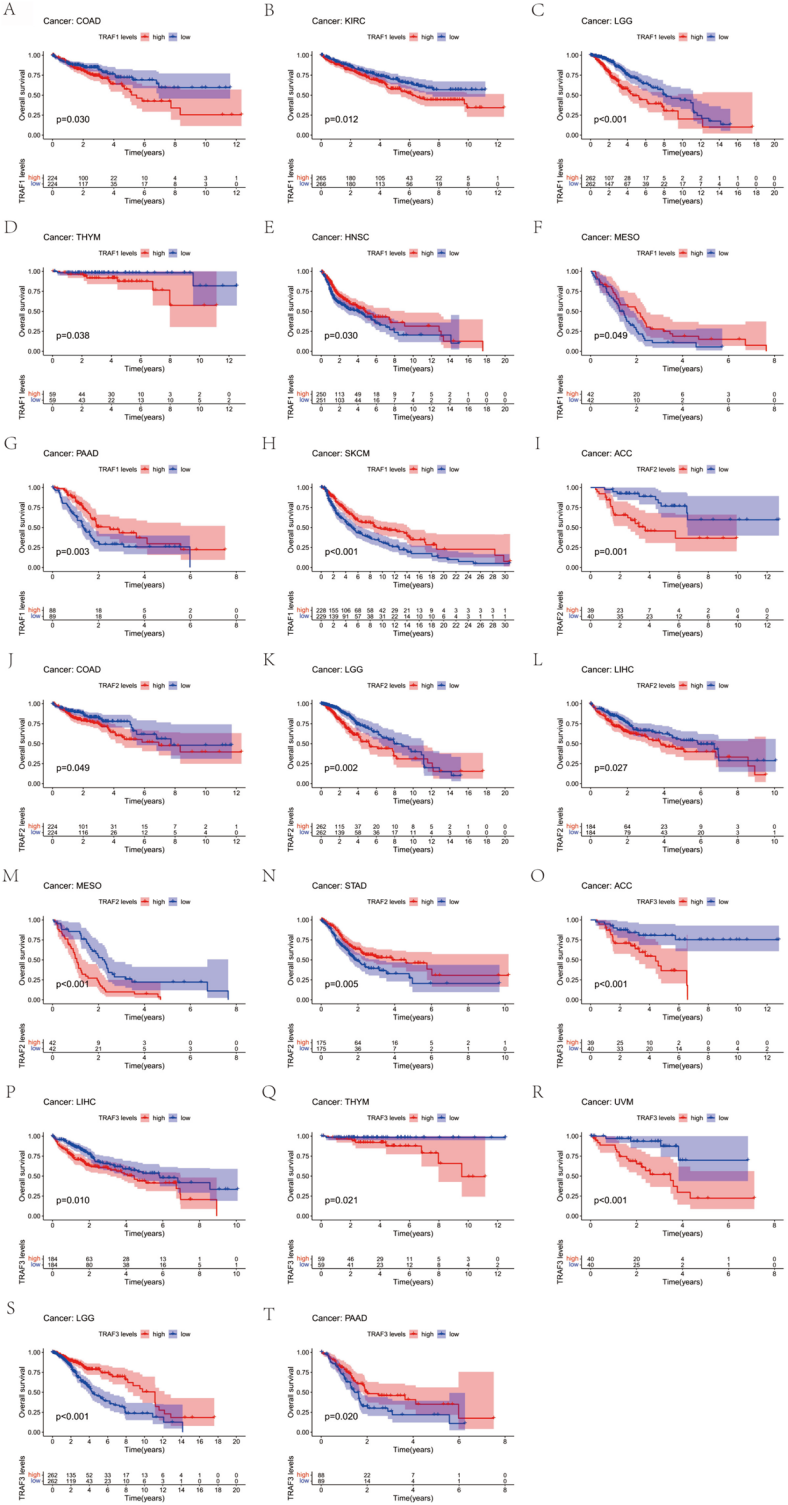
Kaplan–Meier survival curves comparison of high and low expression of TRAF family gene in pan-cancer. OS survival curves of EGFR in different cancers. **A** COAD (OS: *n* = 448, *p* = 0.030). **B** KIRC (OS: *n* = 531, *p* = 0.012). **C** LGG (OS: *n* = 524, *p* < 0.001). **D** THYM (OS: *n* = 118, *p* = 0.038). **E** HNSC (OS: *n* = 501, *p* = 0.030). **F** MESO (OS: *n* = 84, *p* = 0.049). **G** PAAD (OS: *n* = 177, *p* = 0.003). **H** SKCM (OS: *n* = 457, *p* < 0.001). **I** ACC (OS: *n* = 79, *p* = 0.001). **J** COAD (OS: *n* = 448, *p* = 0.049). **K** LGG (OS: *n* = 524, *p* = 0.002). **L** LIHC (OS: *n* = 368, *p* = 0.027). **M** MESO (OS: *n* = 84, *p* < 0.001). **N** STAD (OS: *n* = 350, *p* = 0.005). **O** ACC (OS: *n* = 79, *p* < 0.001). **P** LIHC (OS: *n* = 368, *p* = 0.010). **Q** THYM (OS: *n* = 118, *p* = 0.021). **R** UVM (OS: *n* = 80, *p* < 0.001). **S** LGG (OS: *n* = 524, *p* < 0.001). **T** PAAD (OS: *n* = 177, *p* = 0.020)

To elucidate the prognostic significance of TRAF family genes across various cancers, we conducted a comprehensive analysis utilizing multiple databases. Kaplan–Meier survival analyses revealed a notable association between the expression levels of TRAF family genes and patient prognosis in certain cancers. Specifically, in some cancer types, a trend was observed, where patients with higher TRAF family gene expression exhibited an increased survival rate compared to those with lower expression. Conversely, in other cancer forms, the data indicated that elevated TRAF family gene expression correlated with a decreased survival rate, highlighting a complex and variable impact of these genes on cancer prognosis (Table 1). In this study, TRAF1 was found to function as an oncogene in several cancers. Notably, in COAD (OS: *n* = 448, *p* = 0.030, Fig. 3A), KIRC (OS: *n* = 531, *p* = 0.012, Fig. 3B), Brain Lower Grade Glioma (LGG) (OS: *n* = 524, *p* < 0.001, Fig. 3C), and THYM (OS: *n* = 118, *p* = 0.038, Fig. 3D), elevated expression of TRAF1 was associated with poorer patient outcomes. Conversely, in HNSC (OS: *n* = 501, *p* = 0.030, Fig. 3E), mesothelioma (MESO) (OS: *n* = 84, *p* = 0.049, Fig. 3F), pancreatic adenocarcinoma (PAAD) (OS: *n* = 177, *p* = 0.003, Fig. 3G), and skin cutaneous melanoma (SKCM) (OS: *n* = 457, *p* < 0.001, Fig. 3H), TRAF1 expression correlated with a protective effect, indicating its role as a potential inhibitor in these cancer types. TRAF2 was identified as an oncogenic factor in several cancers. It played a significant role in adrenocortical carcinoma (ACC) (OS: *n *= 79, *p* = 0.001, Fig. 3I), COAD (OS: *n* = 448, *p* = 0.049, Fig. 3J), LGG (OS: *n* = 524, *p* = 0.002, Fig. 3K), LIHC (OS: *n* = 368, *p* = 0.027, Fig. 3L), and MESO (OS: *n* = 84, *p* < 0.001, Fig. 3M). Interestingly, in STAD (OS: *n* = 350, *p* = 0.005, Fig. 3N), TRAF2 exhibited an oncostatic role. Furthermore, TRAF3 demonstrated carcinogenic properties in ACC (OS: *n* = 79, *p* < 0.001, Fig. 3O), LIHC (OS: *n* = 368, *p* = 0.010, Fig. 3P), THYM (OS: *n* = 118, *p* = 0.021, Fig. 3Q), and Uveal Melanoma (UVM) (OS: *n *= 80, *p* < 0.001, Fig. 3R). Conversely, it showed a protective effect in LGG (OS: *n* = 524, *p* < 0.001, Fig. 3S) and PAAD (OS: *n* = 177, *p* = 0.020, Fig. 3T). Finally, TRAF4 was associated with oncogenic effects in ACC (OS: *n* = 79, *p* = 0.003, Supplementary Fig. 2A), HNSC (OS: *n* = 501, *p* = 0.023, Supplementary Fig. 2B), KIRC (OS: *n* = 531, *p* = 0.005, Supplementary Fig. 2C), LIHC (OS: *n* = 368, *p* = 0.032, Supplementary Fig. 2D), and Sarcoma (SARC) (OS: *n* = 260, *p* = 0.036, Supplementary Fig. 2E). In addition, in BLCA (OS: *n* = 406, *p* = 0.015, Supplementary Fig. 2F), TRAF4 also demonstrated oncogenic properties. In this study, TRAF5 was implicated in oncogenic processes in several cancers. Specifically, it contributed to oncogenesis in ACC (OS: *n* = 79, *p* < 0.001, Supplementary Fig. 2G), KIRC (OS: *n* = 531, *p* = 0.001, Supplementary Fig. 2H), KIRP (OS: *n* = 286, *p* = 0.007, Supplementary Fig. 2I), LGG (OS: *n* = 524, *p* < 0.001 Supplementary Fig. 2 J), and LIHC (OS: *n* = 368, *p* = 0.011, Supplementary Fig. 2 K). However, it demonstrated oncostatic effects in BLCA (OS: *n* = 406, *p* = 0.003, Supplementary Fig. 2L) and UCEC (OS: *n* = 544, *p* = 0.028, Supplementary Fig. 2 M). Moreover, TRAF6 was found to exert inhibitory effects on cancer progression in KIRC (OS: *n* = 531, *p* < 0.001, Supplementary Fig. 2N) and READ (OS: *n* = 158, *p* = 0.049, Supplementary Fig. 2O). Finally, TRAF7 was associated with carcinogenic activity in several cancer types, including KIRC (OS: *n* = 531, *p* = 0.003, Supplementary Fig. 2P), LGG (OS: *n* = 524, *p* < 0.001, Supplementary Fig. 2Q), LUSC (OS: *n* = 493, *p* = 0.028, Supplementary Fig. 2R), Ovarian Serous Cystadenocarcinoma (OV) (OS: *n* = 378, *p* = 0.036, Supplementary Fig. 2S), PAAD (OS: *n* = 177, *p* = 0.037, Supplementary Fig. 2 T), and SKCM (OS: *n* = 457, *p* = 0.002, Supplementary Fig. 2U).

**Fig. 4:**
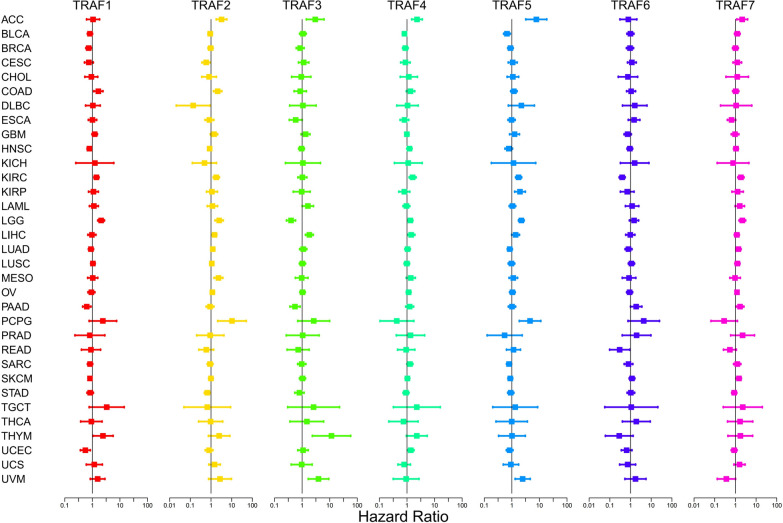
Correlation between TRAF family gene expression and patient survival across various cancer types, as determined by COX regression analysis. It features distinct colored lines, each representing a different TRAF gene. These lines indicate the risk value associated with each gene in different tumor types, hazard ratio < 1 is indicative of a low-risk gene, while HR > 1 signifies a high-risk gene. This visual representation aids in the clear understanding of the complex relationship between TRAF gene expression and cancer prognosis

In our comprehensive analysis utilizing COX regression (Fig. 4), we observed distinct prognostic implications of TRAF family genes across various cancer types. Notably, TRAF1 emerged as a protective prognostic factor in BRCA, HNSC, PAAD, SKCM, and UCEC (HR < 1, *P* < 0.05, Fig. 4, Table 2). Conversely, TRAF1 demonstrated a negative prognostic impact in COAD, KIRC, LGG, and THYM (HR > 1, *P* < 0.05, Fig. 4, Table 2). Moreover, TRAF2 was characterized as a low-risk gene in Cervical squamous cell carcinoma and endocervical adenocarcinoma (CESC), lymphoid neoplasm diffuse large b-cell lymphoma (DLBC), and STAD (HR < 1, *P* < 0.05, Fig. 4, Table 2). Conversely, TRAF2 was identified as a high-risk gene in ACC, COAD, KIRC, LGG, LIHC, MESO, and pheochromocytoma and paraganglioma (PCPG) (HR > 1, *P* < 0.05, Fig. 4, Table 2). TRAF3, on the other hand, was deemed a low-risk gene in LGG and PAAD (HR < 1, *P* < 0.05, Fig. 4, Table 2), while it was classified as a high-risk gene in ACC, Acute Myeloid Leukemia (LAML), LIHC, THYM, and UVM (HR > 1, *P* < 0.05, Fig. 4, Table 2). Furthermore, TRAF4 was recognized as a low-risk gene in BLCA (HR < 1, *P* < 0.05, Fig. 4, Table 2). In contrast, it was noted as a high-risk gene in ACC, KIRC, LGG, LIHC, and UCEC (HR > 1, *P* < 0.05, Fig. 4, Table 2). In addition, TRAF5 was categorized as a low-risk gene in BLCA and SARC (HR < 1, *P* < 0.05, Fig. 4, Table 2), whereas it emerged as a high-risk gene in ACC, KIRC, KIRP, LGG, PCPG, and UVM (HR > 1, *P* < 0.05, Fig. 4, Table 2). In our findings, TRAF6 was characterized as a low-risk gene in KIRC and READ (HR < 1, *P* < 0.05, Fig. 4, Table 2). However, it was identified as a high-risk gene in PAAD (HR > 1, *P* < 0.05, Fig. 4, Table 2). Furthermore, TRAF7 emerged as a high-risk gene across multiple cancer types, specifically in ACC, KIRC, LGG, LUAD, PAAD, and SKCM (HR > 1, *P* < 0.05, Fig. 4, Table 2).

**Table 2:** TRAF family gene was associated with the prognosis risk of different cancers by COX analysis

Gene	Cancer type	HR	HR.95L	HR.95H	p value
TRAF1	BRCA	0.735120785	0.582713166	0.927390353	0.009
	HNSC	0.780767156	0.646642085	0.942712151	0.010
	PAAD	0.629518072	0.447147111	0.886269851	0.008
	SKCM	0.81785633	0.740593068	0.903180176	7.15E-05
	UCEC	0.557218213	0.368679519	0.842173543	0.006
	COAD	1.655353549	1.130475629	2.423931397	0.010
	KIRC	1.40931574	1.139863456	1.74246384	0.002
	LGG	2.083904717	1.575397886	2.756547351	2.68E-07
	THYM	2.439792083	1.050099025	5.668594356	0.038
TRAF2	CESC	0.585229638	0.365362541	0.93740789	0.026
	DLBC	0.137842037	0.021424358	0.886860982	0.037
	STAD	0.662636971	0.478470407	0.917690516	0.013
	ACC	3.236883953	1.790971732	5.85013015	0.000
	COAD	2.09949796	1.327988795	3.31922355	0.002
	KIRC	1.803892534	1.283001588	2.536262078	0.001
	LGG	2.475357305	1.569926006	3.902982536	9.57E-05
	LIHC	1.475983995	1.149450612	1.895278259	0.002
	MESO	2.297296547	1.408728781	3.746336054	0.001
	PCPG	10.32043237	2.107870885	50.53028866	0.004
TRAF3	LGG	0.39577361	0.27159205	0.576735402	1.40E-06
	PAAD	0.554490457	0.363325488	0.846237538	0.006
	ACC	2.979947828	1.424400985	6.23426209	0.004
	LAML	1.613340776	1.009309248	2.578861203	0.046
	LIHC	1.830251475	1.319697829	2.53832384	0.000
	THYM	11.6616549	2.353216539	57.79076966	0.003
	UVM	3.93937307	1.677257548	9.252401457	0.002
TRAF4	BLCA	0.80652504	0.680152728	0.956377315	0.013
	ACC	2.305699779	1.484538011	3.581081407	0.000
	KIRC	1.603190462	1.205204433	2.132600567	0.001
	LGG	1.328845291	1.087211929	1.624181782	0.005
	LIHC	1.422728922	1.048564881	1.930407572	0.024
	UCEC	1.364965061	1.03922768	1.79280215	0.025
TRAF5	BLCA	0.663499108	0.504281471	0.872986796	0.003
	SARC	0.78765713	0.641599232	0.966964615	0.023
	ACC	7.674073261	3.203456615	18.3837047	4.83E-06
	KIRC	1.758185412	1.365394253	2.263973162	1.22E-05
	KIRP	1.971663965	1.264379614	3.074597808	0.003
	LGG	2.200495446	1.736259309	2.788857738	6.86E-11
	PCPG	4.561214934	1.86207341	11.17285794	0.001
	UVM	2.467549437	1.318056207	4.619530028	0.005
TRAF6	KIRC	0.394822474	0.283968318	0.548951329	3.26E-08
	READ	0.298705066	0.097657889	0.913645768	0.034
	PAAD	1.882894779	1.002560264	3.536239046	0.049
TRAF7	ACC	2.135496469	1.172413454	3.889707298	0.013
	KIRC	1.850576665	1.324005291	2.58657123	3.15E-04
	LGG	2.168237968	1.506590267	3.120460811	3.09E-05
	LUAD	1.406061055	1.053360729	1.87685722	0.021
	PAAD	1.672451912	1.090504561	2.564955249	0.018
	SKCM	1.479749685	1.135447536	1.928454693	0.004

**Fig. 5:**
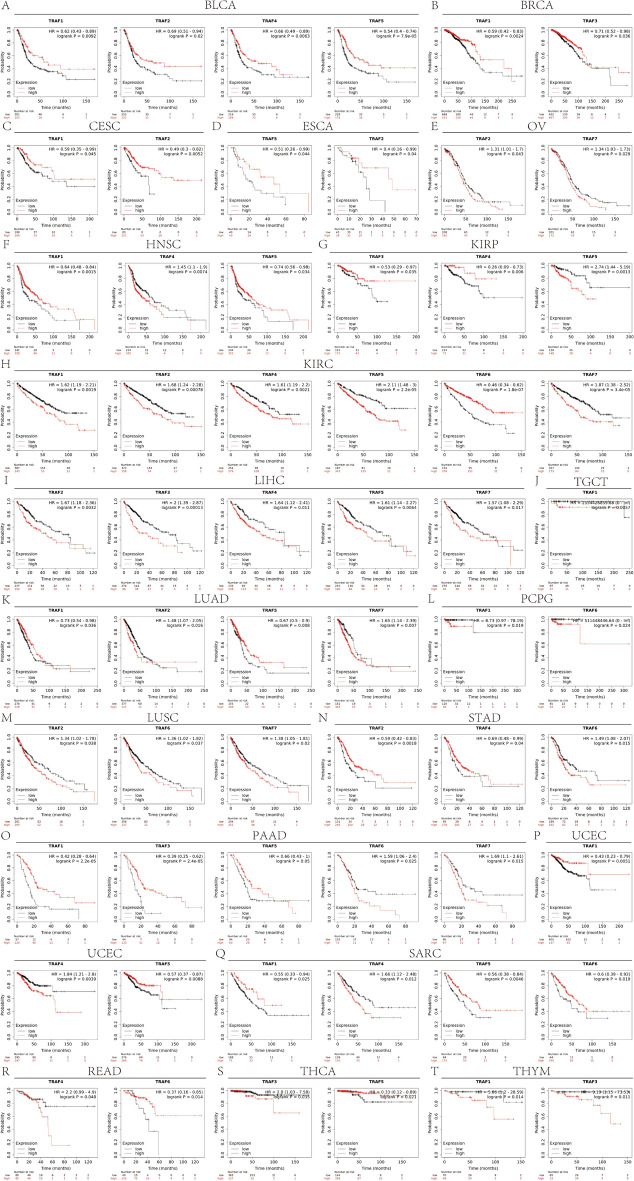
Overall survival curves comparing the high and low expression of TRAF family gene in various cancer types in Kaplan–Meier plotter database. **A** TRAF1, TRAF2, TRAF4 and TRAF5 in BLCA. **B** TRAF1and TRAF3 in BRCA. **C** TRAF1 and TRAF2 in CESC. **D** TRAF2 and TRAF5 in ESCA. **E** TRAF2 and TRAF7 in OV. **F** TRAF1, TRAF4, and TRAF5 in HNSC. **G** TRAF3, TRAF4 and TRAF5 in KIRP. **H** TRAF1, TRAF2, TRAF4, TRAF5, TRAF6 and TRAF7 in KIRC. **I** TRAF2, TRAF3, TRAF4, TRAF5 and TRAF7 in LIHC. **J** TRAF1 in TGCT. **K** TRAF1, TRAF2, TRAF5 and TRAF7 in LUAD. **L** TRAF1 and TRAF6 in PCPG. **M** TRAF2, TRAF6 and TRAF7 in LUSC. **N** TRAF2, TRAF4 and TRAF6 in STAD. **O** TRAF1, TRAF3, TRAF5, TRAF6 and TRAF7 in PAAD. **P** TRAF1, TRAF4 and TRAF5 in UCEC. **Q** TRAF1, TRAF4, TRAF5 and TRAF6 in SARC. **R** TRAF4 and TRAF6 in READ. **S** TRAF3 and TRAF5 in THCA. **T** TRAF1 and TRAF3 in THYM

To further delineate the prognostic significance of TRAF family genes in a pan-cancer context, we conducted a validation study using the Kaplan–Meier plotter. This analysis reinforced our findings, revealing that TRAF1, TRAF2, TRAF4, and TRAF5 play an inhibitory role in BLCA (Fig. 5A). In addition, TRAF1 and TRAF3 were identified as suppressor oncogenes in BRCA (Fig. 5B). In the context of CESC, both TRAF1 and TRAF2 emerged as suppressor oncogenes (Fig. 5C). For ESCA, TRAF2 and TRAF5 were recognized as suppressor genes (Fig. 5D). Finally, TRAF2 and TRAF7 were found to inhibit OV (Fig. 5E). In the context of HNSC, our findings indicate that TRAF1 and TRAF5 act as tumor suppressors, whereas TRAF2 emerges as an oncogenic factor (Fig. 5F). In KIRP, both TRAF3 and TRAF4 are identified as carcinogenic factors, contrastingly, TRAF5 functions as an inhibitor (Fig. 5G). Within KIRC, our analysis shows that TRAF1, TRAF2, TRAF4, TRAF5, and TRAF7 play oncogenic roles, while TRAF6 appears to serve as an oncostatic factor (Fig. 5H). In the case of LIHC, TRAF2, TRAF3, TRAF4, TRAF5, and TRAF7 are identified as suppressor genes (Fig. 5I). In addition, patients with heightened TRAF1 expression in TGCT are associated with a poorer prognosis compared to those with lower expression levels (Fig. 5J). In LUAD, our analysis identified TRAF2 and TRAF7 as oncogenic factors, whereas TRAF1 and TRAF6 were found to play oncostatic roles (Fig. 5K). Regarding PCPG, TRAF1 and TRAF6 were determined to be carcinogenic factors (Fig. 5L). In LUSC, TRAF2, TRAF6, and TRAF7 were recognized as inhibitory factors (Fig. 5M). In STAD, patients exhibiting high TRAF6 expression were associated with a worse prognosis, while those with elevated TRAF2 and TRAF4 expressions had a more favorable prognosis compared to individuals with low expression levels (Fig. 5N). In PAAD, TRAF2 and TRAF7 were characterized as carcinogenic, in contrast, TRAF1, TRAF3, and TRAF5 acted as inhibitors (Fig. 5O). In the case of UCEC, a dismal prognosis was associated with high TRAF4 expression, whereas patients with high expressions of TRAF1 and TRAF5 had better prognoses compared to those in the low-expression group (Fig. 5P). In patients diagnosed with SARC, elevated expression levels of TRAF4 correlate with an unfavorable prognosis. Conversely, increased levels of TRAF1, TRAF5, and TRAF6 are associated with a more favorable prognosis, in contrast to patients exhibiting lower expression levels of these factors (Fig. 5Q). Similarly, in READ cases, a high TRAF4 expression is indicative of an adverse prognosis. On the other hand, elevated TRAF6 expression suggests a more positive outcome when compared to patients with lower expression levels of this factor (Fig. 5R). Furthermore, in THCA, a high expression of TRAF3 is linked to a detrimental prognosis, whereas increased TRAF5 expression correlates with a better prognosis relative to those with lower expression levels (Fig. 5S). Finally, in THYM patients, heightened expression of both TRAF1 and TRAF3 is associated with a less favorable prognosis when compared to patients with lower expression levels of these factors (Fig. 5T).

In our comprehensive analysis, we explored the association between TRAF family gene expression and overall cancer prognosis using the PrognoScan online database. This database compiles data sourced from the GEO database. For a detailed exposition of these findings, refer to Table S2. Upon examining the outcomes from various databases, a spectrum of findings emerged regarding the TRAF family's expression in diverse cancer types, as summarized in Table 3. While some discrepancies were noted, a portion of the data exhibited consistency. These variations in findings may stem from differences in data collection methodologies and underlying theoretical frameworks that account for distinct biological characteristics of each cancer type. Notwithstanding these discrepancies, the congruent data present a more robust indication of the TRAF family's prognostic significance in oncology.

**Table 3:** Association between TRAF family gene high expression and pan-cancer in different database

Gene	Role	TCGA (Kaplan Meier)	TCGA (COX)	Kaplan–Meier plotter
TRAF1	Detrimental	KIRC, THYM, COAD, LGG	KIRC, THYM, COAD, LGG	KIRC, THYM, TGCT, PCPG
	Protective	HNSC, PAAD, SKCM, MESO	HNSC, PAAD, BRCA, SKCM, UCEC	HNSC, PAAD, BLCA, BRCA, CESC, LUAD, UCEC, SARC
TRAF2	Detrimental	LIHC, ACC, COAD,LGG,MESO	LIHC, ACC, COAD,KIRC,LGG,MESO,PCPG	LIHC,OV,KIRC, LUAD,LUSC
	Protective	STAD	STAD, CESC, DLBC	STAD, BLCA, CESC, ESCA
TRAF3	Detrimental	LIHC, THYM, ACC, UVM	LIHC, THYM, ACC, LAML, UVM	LIHC,THYM, THCA
	Protective	PAAD, LGG	PAAD, LGG	PAAD, BRCA, KIRP
TRAF4	Detrimental	KIRC, LIHC, ACC, HNSC, SARC	KIRC, LIHC, ACC, LGG, UCEC	KIRC, LIHC, HNSC, UCEC, SARC, READ
	Protective	BLCA	BLCA	BLCA, KIRP, STAD
TRAF5	Detrimental	KIRC, KIRP, ACC, LGG, LIHC	KIRC, KIRP, ACC, LGG, PCPG, UVM	KIRP, KIRC, LIHC
	Protective	BLCA, UCEC	BLCA, SARC	BLCA, ESCA, HNSC, LUAD, PAAD, UCEC, SARC, THCA
TRAF6	Detrimental		PAAD	PCPG, LUSC, STAD, PAAD
	Protective	KIRC, READ	KIRC, READ	KIRC, READ, SARC
TRAF7	Detrimental	KIRC, PAAD, LGG, LUSC, OV, SKCM	KIRC, PAAD, ACC, LGG, LUAD, SKCM	KIRC, PAAD, LIHC, LUAD, LUSC, OV
	Protective			

### Association of TRAF family gene expression with TME, stemness score, and immune subtypes in pan-cancer and selected cancers

**Fig. 6:**
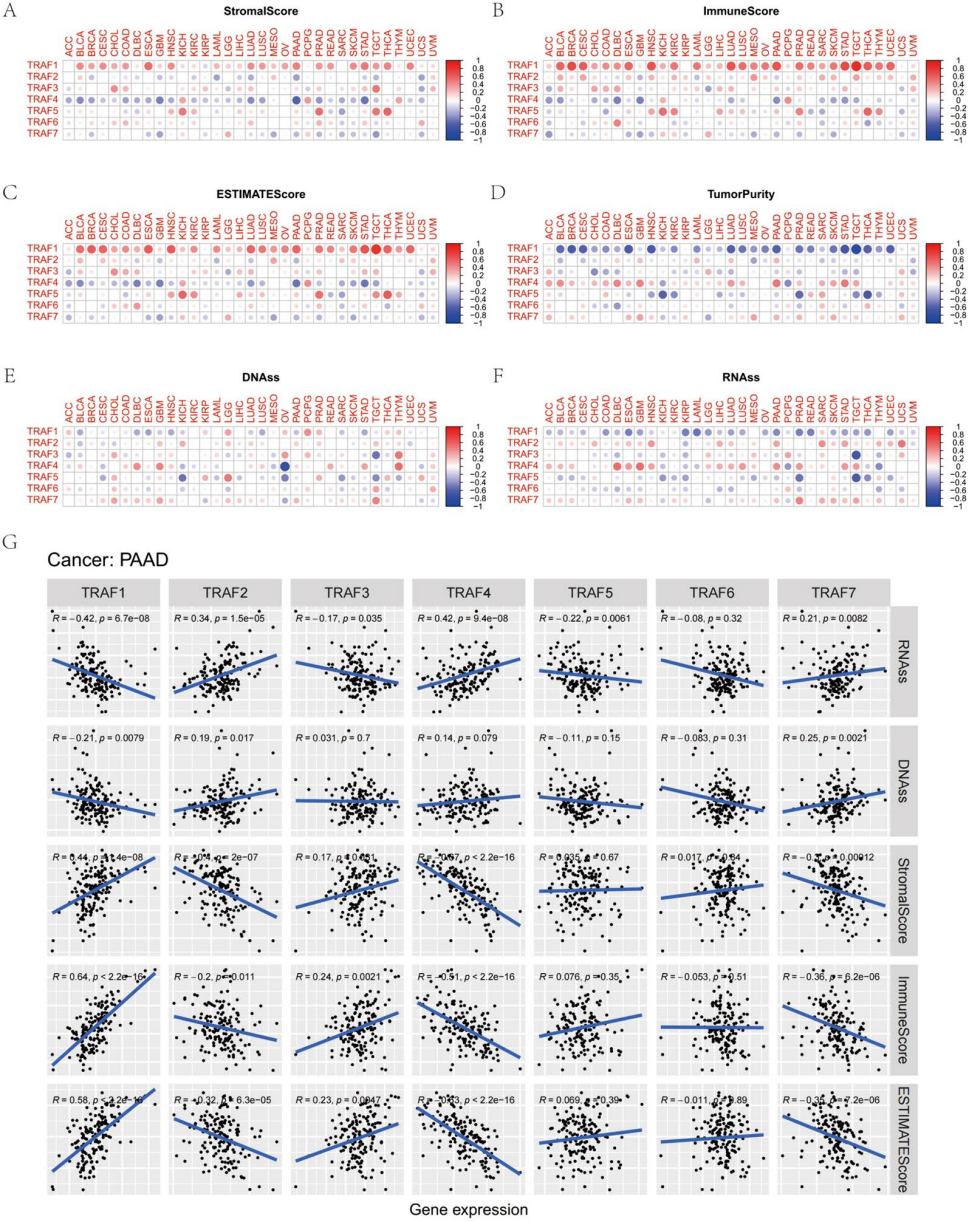
Correlation of TRAF family gene expression with tumor microenvironment, Stemness score in pan-cancer. **A, B, C, D** TRAF family gene expression associated with stromal score, immune score, ESTIMAT score and tumour purity in different cancers. **E, F** TRAF family gene expression associated with DNAss and RNAss in different cancers. Red dots indicate a positive correlation, and blue dots indicate a negative correlation. **G** Correlation analysis of TRAF family gene expression with Stemness score, tumor microenvironment in PAAD

The TME plays a critical role in tumor development, metastasis, and response to therapy. In this context, we investigated the relationship between TRAF family gene expression and various aspects of the TME. We utilized the ESTIMATE method to derive stromal and immune scores, and also calculated tumor purity, as detailed in Table S3. Our analysis revealed that TRAF family gene expression demonstrates significant positive or negative correlations with both stromal (Fig. 6A) and immune (Fig. 6B) scores across a range of cancers. In a pan-cancer analysis, the expression of TRAF family genes exhibited significant correlations, both positive and negative, with RNAss (Fig. 6C) and DNAss (Fig. 6D). Specifically, within PAAD, TRAF1 and TRAF3 demonstrated positive correlations with stromal, immune, and estimated scores while showing negative correlations with tumor purity. Conversely, TRAF2, TRAF4, and TRAF7 displayed positive correlations with stromal, immune, and estimated scores, and negative correlations with tumor purity. Furthermore, a negative correlation of TRAF1 with DNAss was observed, in contrast to the positive correlations of TRAF2 and TRAF7. In addition, TRAF1, TRAF3, and TRAF5 showed negative correlations with RNAss, whereas TRAF2, TRAF4, and TRAF7 exhibited positive correlations.

**Fig. 7:**
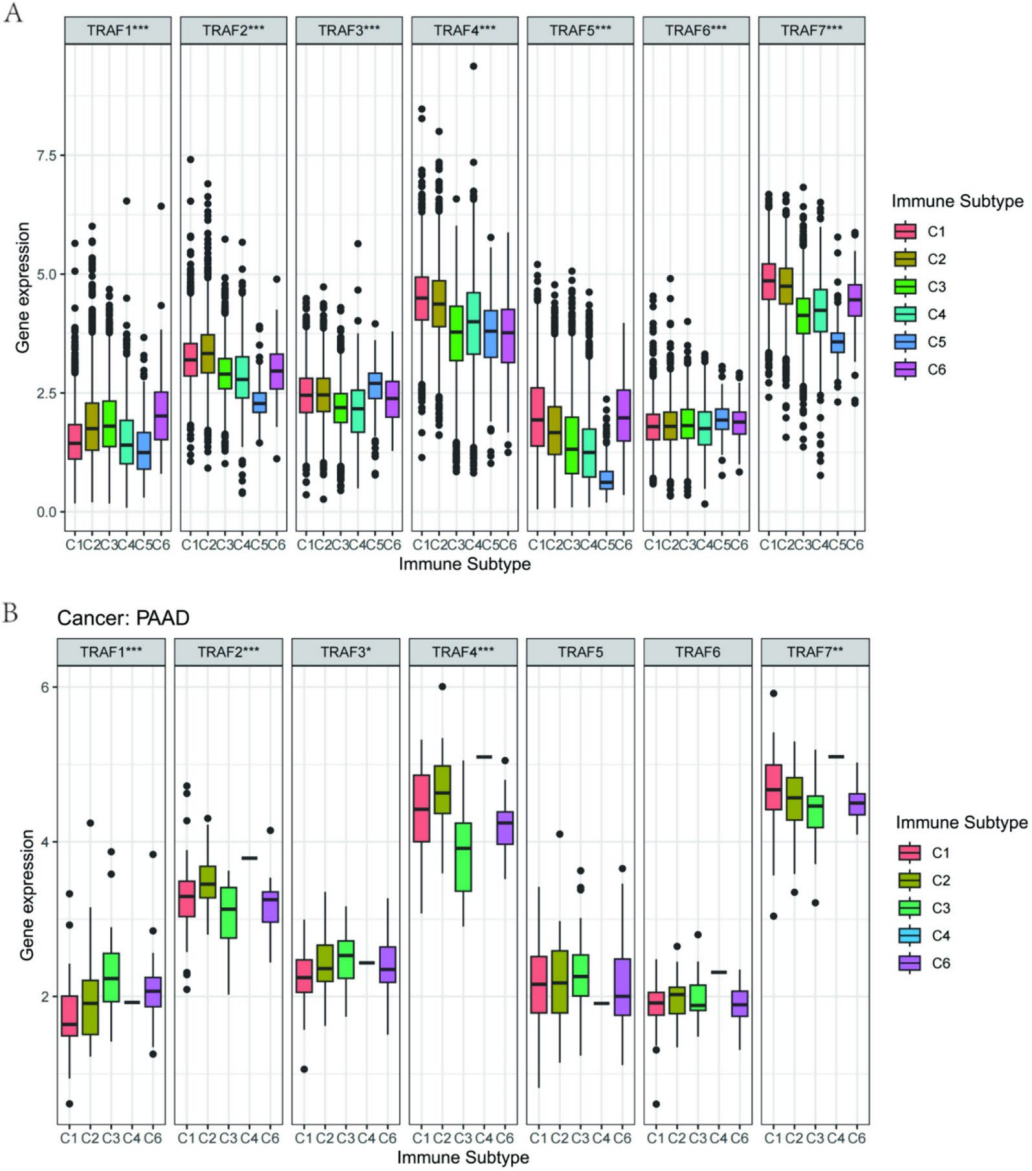
TRAF family gene expression level of different immune subtype in pan-cancer and KIRC. **A** All seven genes of the TRAF family were associated with immune subtypes in pan-cancer. **B** TRAF family gene expression level in different immune subtypes in PAAD

In their comprehensive immunogenomic study, Thorsson et al. analyzed over 10,000 tumors encompassing 33 distinct cancer types from the TCGA database. This analysis led to the identification of six immune subtypes: wound-healing (C1), IFN-γ dominant (C2), inflammatory (C3), lymphocyte depleted (C4), immunologically quiet (C5), and TGF-β dominant (C6) [18]. These subtypes have significant implications for both prognosis and immune regulation. Building upon this foundation, this study investigated the association between TRAF family genes and these immune subtypes. Our findings reveal that all seven TRAF family genes are correlated with specific immune subtypes (Fig. 7A). Within the context of PAAD, we discovered associations between immune subtypes and five TRAF genes, namely TRAF1, TRAF2, TRAF3, TRAF4, and TRAF7 (Fig. 7B).

### Correlation between the expression of TRAF family genes and drug sensitivity

**Fig. 8:**
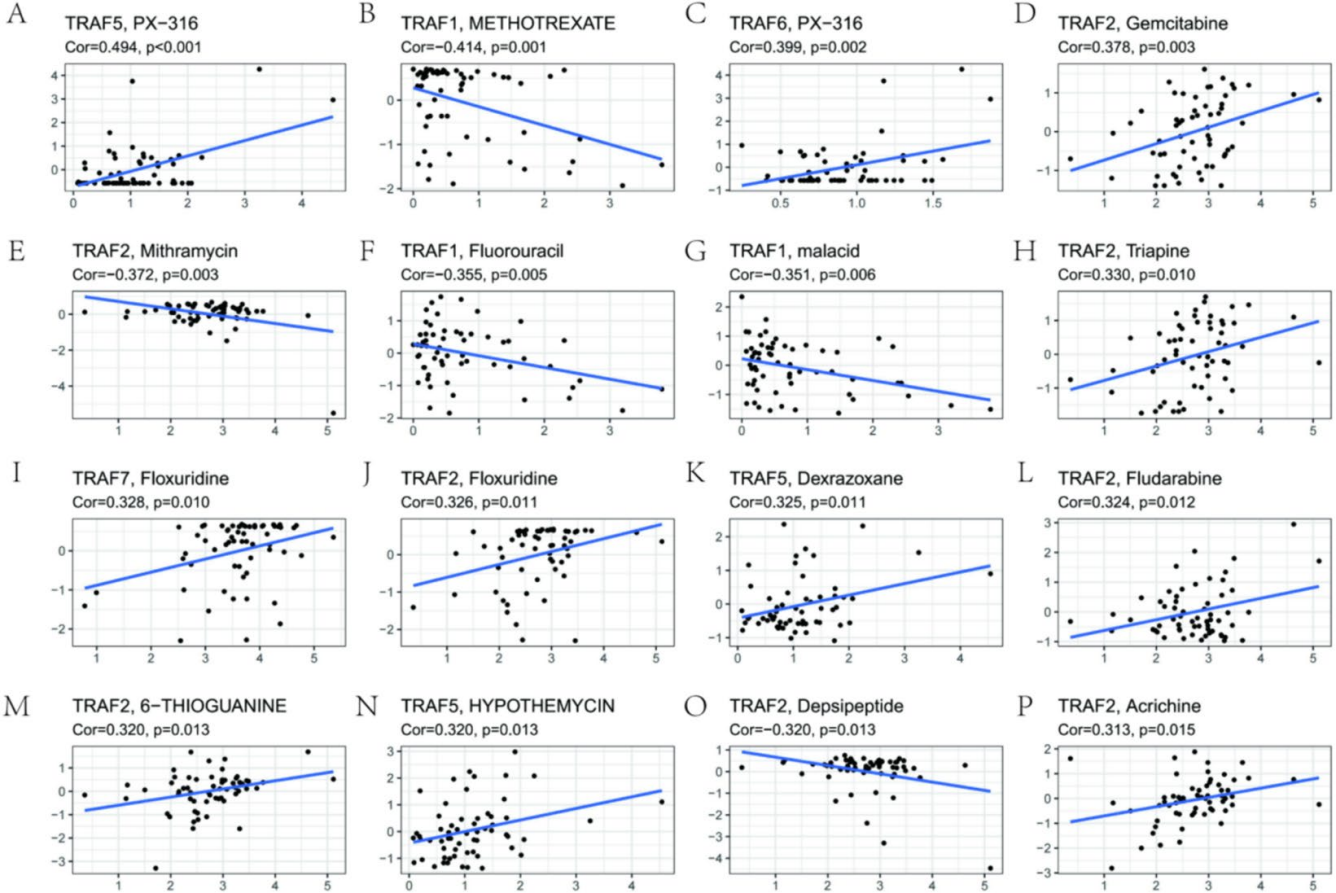
Correlation between the expression of TRAF family genes and drug sensitivity. TRAF1 is negatively correlated with sensitivity of METHOTREXATE, Fluorouracil and malacid **B, F, G**. TRAF2 was positively correlated with Gemcitabine, Triapine, Floxuridine, Fludarabine, 6-THIOGUANINE and Acrichine sensitivity **D, H, J, L, P**, but negatively correlated with Mithramycin and Depsipeptide sensitivity **E, O**. TRAF5 was positively correlated with PX-316, Dexrazoxane and HYPOTHEMYCIN sensitivity (A, K, N). TRAF6 was positively correlated with the sensitivity of PX-316 (**C**). TRAF7 was positively correlated with Floxuridine sensitivity (**I**)

We explore the relationship between TRAF family gene expression and drug sensitivity. Utilizing the CellMiner database, we accessed sensitivity data for FDA-approved drugs and those currently in clinical trials. Our analysis demonstrates a distinct correlation between TRAF family gene expressions and drug sensitivity, as depicted in Fig. 8 and Table S4. Specifically, TRAF1 shows a negative correlation with the sensitivity of METHOTREXATE, Fluorouracil, and Malacid (Fig. 8B, F, G). In contrast, TRAF2 exhibits a positive correlation with the sensitivity of Gemcitabine, Triapine, Floxuridine, Fludarabine, 6-THIOGUANINE, and Acrichine (Fig. 8D, H, J, L, P), but a negative correlation with Mithramycin and Depsipeptide (Fig. 8E, O). TRAF5 correlates positively with the sensitivity of PX-316, Dexrazoxane, and Hypothemycin (Fig. 8A, K, N). TRAF6 is positively correlated with PX-316 sensitivity (Fig. 8C), and TRAF7 shows a positive correlation with Floxuridine sensitivity (Fig. 8I).

### Expression of TRAF family genes in PAAD

**Fig. 9:**
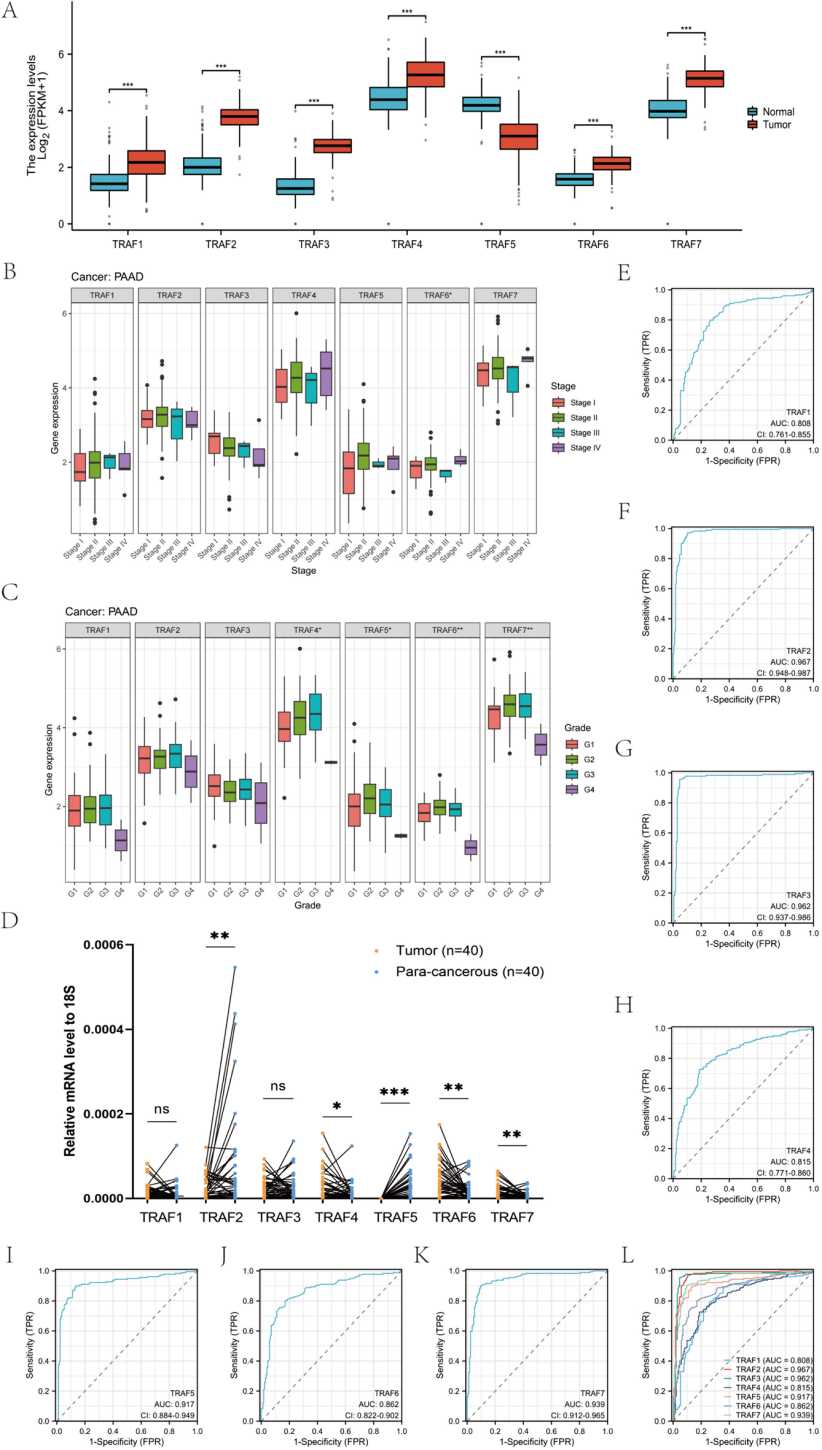
TRAF family genes in PAAD. **A** Expression level of TRAFs in TCGA–PAAD and GTEx-pancreas. **B** TRAF6 was differentially expressed in the TNM stage of PAAD. **C** TRAF4, TRAF5, TRAF6, and TRAF7 were differentially expressed in the degree of differentiation of PAAD. **D** mRNA expression of TRAF genes in 40 PAAD tissues and para-cancerous tissues. **E–L** Diagnostic value analysis of TRAFs. ns: not statistically significant, *: *p* < 0.05, **: *p* < 0.01, ***: *p* < 0.001

Leveraging data from the TCGA database for pancreatic cancer and corresponding normal tissue data sourced from GTEx, extracted via the UCSC database, we conducted a comparative analysis. This analysis revealed that TRAF1, TRAF2, TRAF3, TRAF4, TRAF6, and TRAF7 exhibit higher expression levels in cancer tissues, whereas TRAF5 is expressed at lower levels in these tissues (Fig. 9A). Furthermore, our correlation analysis with clinicopathological features indicates a significant association of TRAF6 expression with the TNM stage of pancreatic cancer. In addition, TRAF4, TRAF5, TRAF6, and TRAF7 demonstrate correlations with the degree of pancreatic cancer differentiation (Fig. 9B, C).

In addition, we employed qRT–PCR to validate the expression levels of TRAF family genes in PAAD. Analyzing 40 pairs of PAAD and adjacent non-tumorous tissues, our findings indicate that the relative mRNA expression levels of TRAF4, TRAF6, and TRAF7 are elevated in PAAD tissues. Conversely, TRAF2 and TRAF5 exhibit higher expression in adjacent non-tumorous tissues (Fig. 9D). Notably, the expression levels of TRAF1 and TRAF3 in PAAD tissues do not significantly differ from those in adjacent non-tumorous tissues (Fig. 9D). These observations align closely with the results discussed in the previous sections.

Furthermore, this study conducted an in-depth evaluation of the diagnostic potential of TRAF family genes in pancreatic cancer. Data for TCGA–PAAD and normal pancreatic tissues from GTEx were downloaded from the UCSC XENA platform. We employed ROC analysis using the "pROC" package in R and visualized the results with "ggplot2". The analysis reveals that all TRAF family genes exhibit high accuracy in differentiating between normal and tumor tissues in pancreatic cancer, as evidenced by the ROC curves presented in Fig. 9E–L.

TRAF6 promotes proliferation, migration and invasion of pancreatic cancer cells in vitro.

**Fig. 10:**
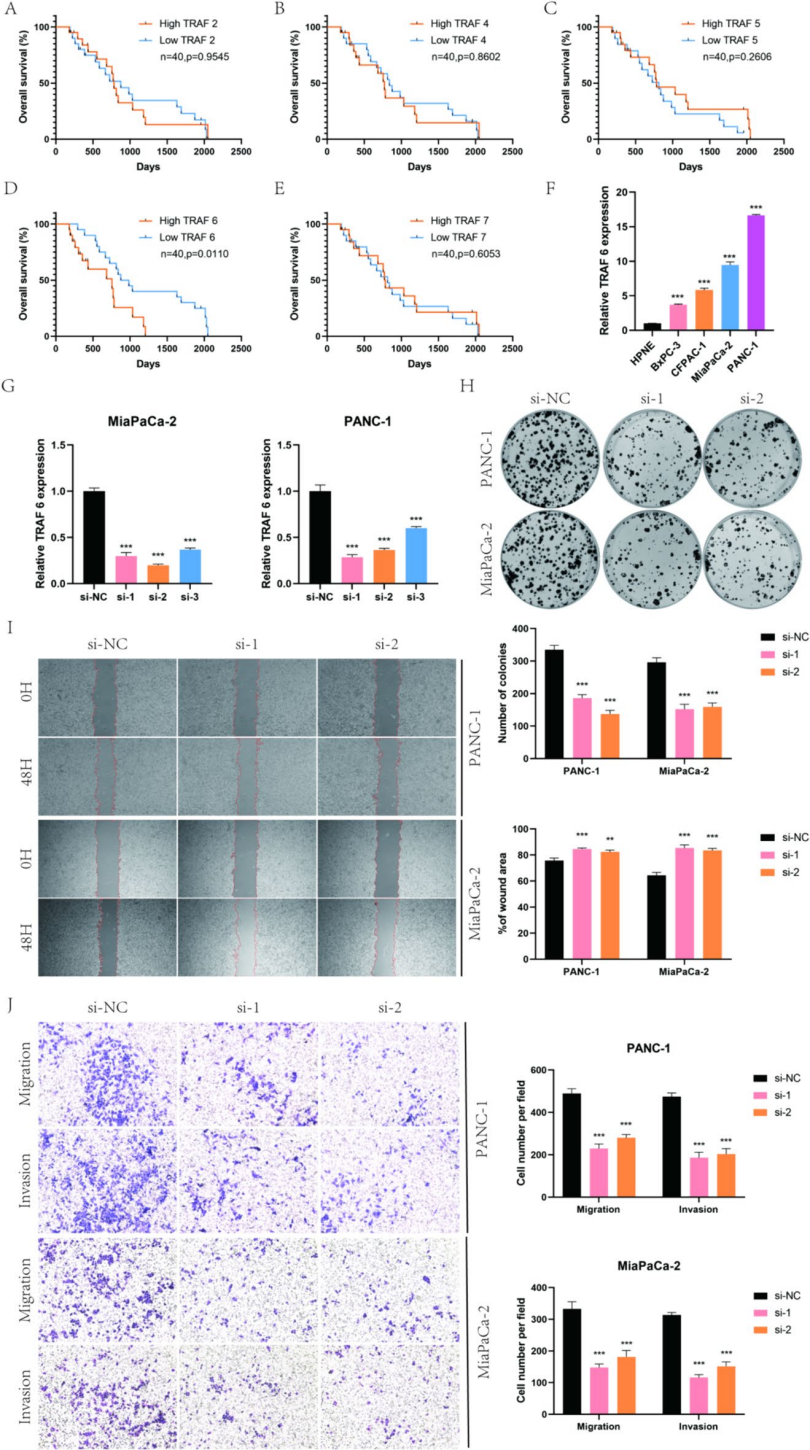
TRAF6 promotes the proliferation, migration and invasion of PDAC cells in vitro. **A–E** Kaplan–Meier survival curves comparison of high and low expression of TRAFs in pancreatic cancer. **F** Expression of TRAF6 in PDAC cells. **G** qRT–PCR analysis of TRAF6 in PANC-1 and MiaPaCa-2 cells transfected with TRAF6 siRNAs. **H** Colony formation assays were performed in PDAC cells transfected with TRAF6 siRNAs. **I, J** Wound healing assays and Transwell assays were used to investigate the migratory and invasive ability of TRAF6-knockdown PDAC cells. (Values are expressed as the means ± SDs; *P < 0.05, **P < 0.01 and ***P < 0.001)

To further explore the role of TRAFs in pancreatic cancer, we first verified the correlation between TRAFs expression and prognosis in 40 pairs of pancreatic cancer tissue samples. The results showed that only TRAF6 was associated with pancreatic cancer prognosis (Fig. 10A–E). Subsequently, we examined TRAF6 expression in HPNE and PDAC cell lines (BxPC-3, CFPAC-1, MiaPaCa-2, and PANC-1). We found that TRAF6 was highly expressed in PDAC cells, and PANC-1 and MiaPaCa-2 cell lines were used for further studies (Fig. 10F). To assess the biological function of TRAF6 in PDAC cells, we designed and synthesized short interfering RNA to knockdown TRAF6 expression and verified the effectiveness of TRAF6 knockdown by qRT–PCR (Fig. 10G). Clone formation experiments showed that down-regulation of TRAF6 inhibited proliferation (Fig. 10H). In addition, the results of wound healing assay and transwell assay showed that knockdown of TRAF6 significantly reduced the migration and invasion ability of PDAC cells (Fig. 10I, J). Taken together, these observations illustrate the oncogenic role of TRAF6 in PDAC cells.

## Discussion

A hallmark of cancer is its pronounced cell proliferation, migration, invasion, and metastasis. Supporting these processes, a multitude of gene mutations and signaling pathways are implicated. Within this complex network, TRAF family genes play a critical role in tumorigenesis and metastasis. They function as adapters or E3 ubiquitin ligases in various signaling pathways, contributing to the intricate molecular mechanisms underlying cancer progression [19–22]. The existing literature demonstrates a notable correlation between the expression of TRAF family genes and clinicopathological features across a wide range of cancers [23–26]. This evidence suggests that TRAF family genes hold potential as diagnostic and prognostic biomarkers for various tumors. Furthermore, studies have shown that modulating the expression of TRAF family genes, either through inhibition or enhancement, can significantly impact tumor progression both in vitro and in vivo [27]. Consequently, these genes are emerging as promising targets for the prevention and treatment of diverse cancer types. Recent advancements in the study of TRAF family genes have highlighted their potential as therapeutic targets in certain cancers, underscoring their significant role in tumor development and progression. For instance, TRAF1 is found to be underexpressed in renal cell carcinoma. Experimental evidence indicates that reducing TRAF1 expression can exacerbate the proliferation of renal cell carcinoma cells, diminish treatment-induced apoptosis, and increase resistance to Sunitinib, a key therapeutic agent [28, 29]. In gastric cancer, overexpression of TRAF2 has been associated with a poorer prognosis. This is attributed to the activation of the NF-κB pathway and increased IL-8 expression, which collectively contribute to the enhanced invasion and metastasis of gastric cancer cells [30]. In tumor samples, as compared to normal samples, a notable decrease in the expression level of TRAF3 has been observed, implying a potential inhibitory role of TRAF3 in the onset and progression of breast cancer. Furthermore, higher TRAF3 expression is positively correlated with extended relapse-free survival (RFS), OS, and distant metastasis-free survival (DMFS) in breast cancer patients [31]. In the context of HGSOC, TRAF4 is linked to poorer patient prognosis. It appears to promote the malignant progression of HGSOC by activating the YAP pathway [32]. TRAF6 exhibits high expression levels in breast cancer, particularly in cases with bone and brain metastases, and its expression is inversely correlated with breast cancer prognosis. Strategies aimed at reducing TRAF6 expression have been shown to inhibit the proliferation and metastasis of breast cancer cells [33]. These findings collectively underscore the intimate link between TRAF family genes and tumor development. Given their significant influence on cancer progression, TRAF family genes represent promising therapeutic targets for improving tumor prognosis.

Our research reveals that the seven genes constituting the TRAF family exhibit varied effects across different tumor types. Specifically, TRAF2, TRAF4, and TRAF7 are generally overexpressed in a pan-cancer context, and high expression levels of these genes are associated with poorer patient prognosis. This suggests an oncogenic role for these three genes in various tumors, aligning with earlier studies on breast, gastric, and pancreatic cancers [30, 34–36]. These findings highlight the differential impact of TRAF family genes in cancer and underscore their potential as targets for therapeutic intervention. Moreover, this study establishes correlations between TRAF family genes and various facets of the TME, immune subtypes, and drug sensitivity. Notably, TRAF2 and TRAF7 demonstrate a significant positive correlation with the chemotherapeutic agents fluorouracil and gemcitabine, which are commonly used in the treatment of pancreatic cancer. This finding is in line with previous research. Furthermore, we verified the expression levels of TRAF family genes in pancreatic cancer tissues. Data from online databases indicated that TRAF5 was the only gene expressed at lower levels in pancreatic cancer tissues. These results were corroborated by the qRT–PCR analyses conducted in our validation cohort, confirming the consistency of the findings across different methodologies. In addition, this study included a preliminary assessment of the diagnostic potential of TRAF family genes in pancreatic cancer. The findings from this assessment indicated that TRAF2, TRAF3, TRAF5, and TRAF7 are effective in predicting the outcomes of pancreatic cancer. This suggests that these genes could be valuable biomarkers for the diagnosis of this malignancy, contributing to earlier detection and potentially improved patient prognoses. However, in our cohort of 40 pancreatic cancer samples, only TRAF6 showed a correlation with prognosis. This may be due to the small sample counts. To further explore the role of TRAF6 in pancreatic cancer, we knocked down TRAF6 in PDAC cells and found that the proliferation, migration, and invasion of PDAC cells were decreased, suggesting that TRAF6 promotes pancreatic cancer progression.

While this study conducted a comprehensive pan-cancer analysis of TRAF family genes, examining their correlations with survival, the TME, and therapeutic targets, it is important to acknowledge certain limitations. Primarily, the study was centered on bioinformatics analysis of the expression of TRAF family genes and their association with survival prognosis, supplemented by in vitro experimental validation. Future research should delve deeper into the mechanisms of TRAF family genes at the cellular and molecular levels. Such investigations are crucial to fully understand the roles of these genes in various cancer types, particularly in elucidating the underlying mechanisms of the positive findings observed in this study.

## Conclusions

In summary, our research has effectively highlighted the significance of TRAF family genes as diagnostic, therapeutic, and prognostic markers in the context of pan-cancer. This study underscores the potential of developing drug therapies targeting TRAF family genes, offering a promising strategy for cancer treatment.

### Supplementary Information

Supplementary Material 1: Figure 1: Differential expression of TRAF 4–7 in pan-carcinoma and para-carcinoma.

Supplementary Material 2: Figure 2: Prognostic value of TRAF 4–7 in pan-cancer.

Supplementary Material 3: Table S1: Detailed information on 33 cancers.

Supplementary Material 4: Table S2: TRAF family gene expression was related to the prognosis of different cancers in PrognoScan.

Supplementary material 5: Table S3: Correlation coefficients and *p* values for the association between TRAF family and ESTIMATE scores, RNAss, and DNAss.

Supplementary Material 6: Table S4: Correlation coefficients and *p* values for the drug sensitivity analysis of TRAF family genes

Supplementary material 7: Table S5: Sequences of primers and siRNAs used in this study

## Data Availability

The original contributions of this study are included in the article and Supplementary Material. For further information, inquiries can be directed to the corresponding authors.
