# Supplementary figures and images for: Pan-cancer analysis of the TRAF family genes and their correlation with prognosis, TME, immune and drug sensitivity
Source: Eur J Med Res. 2024 Jun 2;29:307. doi: 10.1186/s40001-024-01875-8 (PMC11145793; doi:10.1186/s40001-024-01875-8)

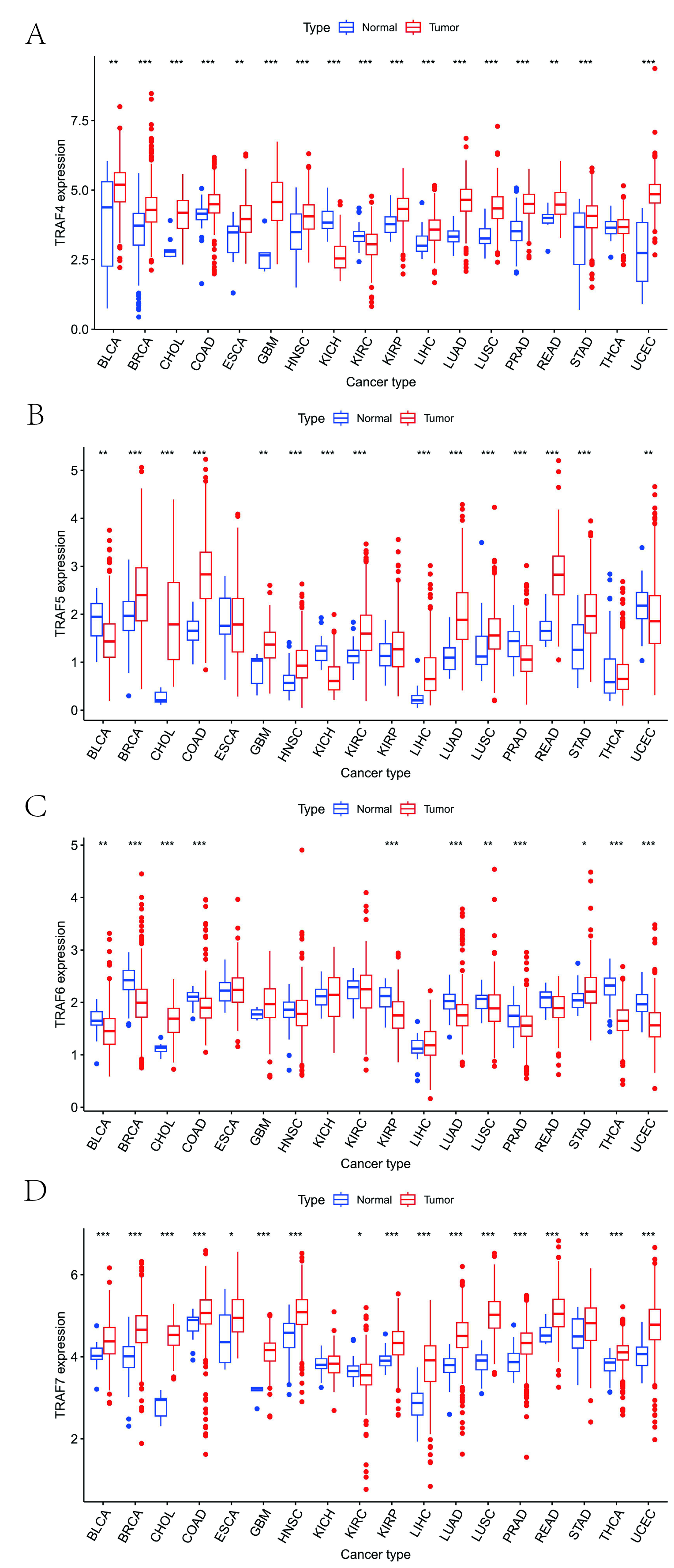

Supplement: Supplementary file 1 — Supplementary Material 1: Figure 1: Differential expression of TRAF 4–7 in pan-carcinoma and para-carcinoma. [file 40001_2024_1875_MOESM1_ESM.tif]

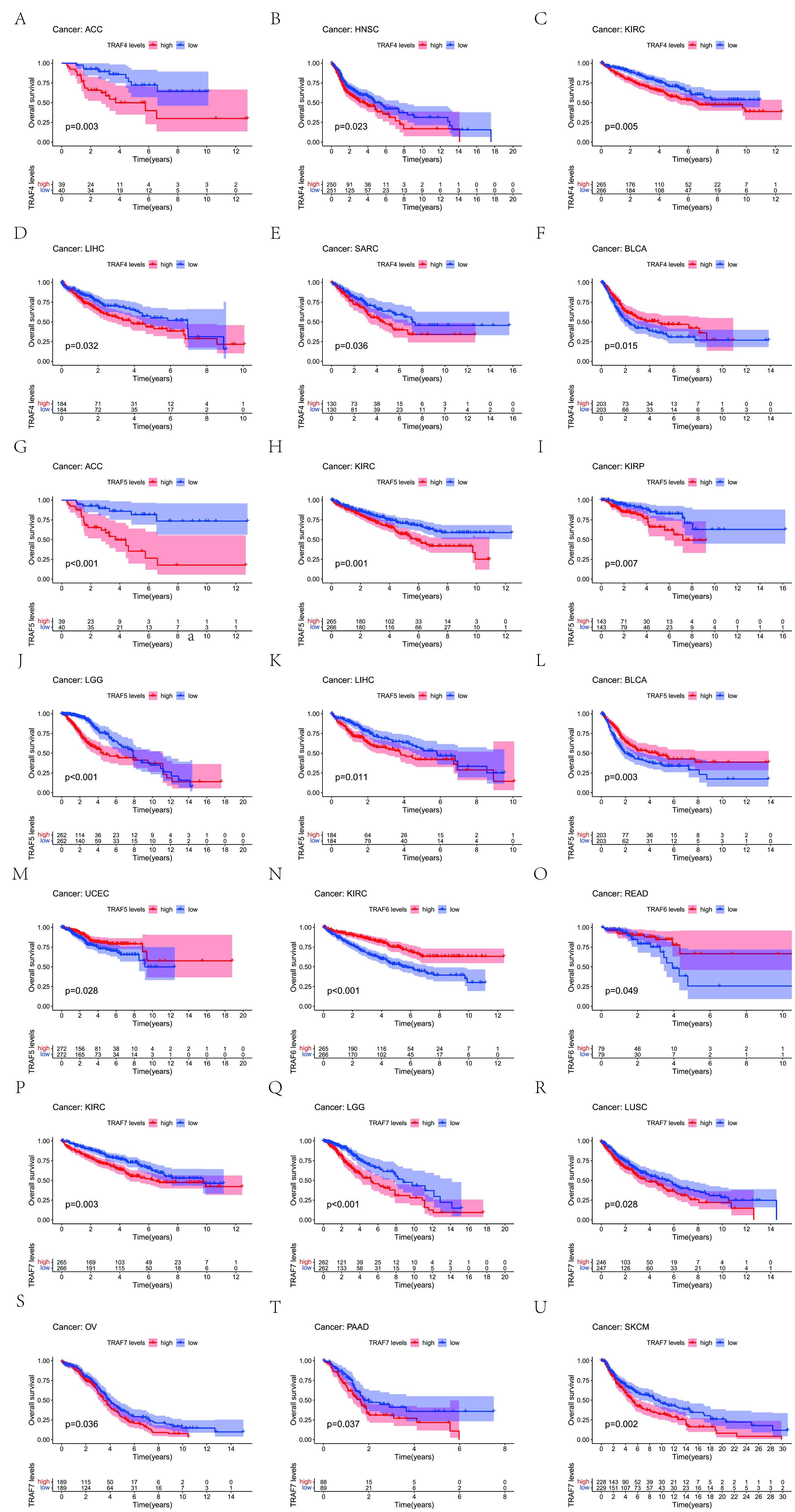

Supplement: Supplementary file 2 — Supplementary Material 2: Figure 2: Prognostic value of TRAF 4–7 in pan-cancer. [file 40001_2024_1875_MOESM2_ESM.tif]
